# Introducing cancers of the head and neck, a new open access journal

**DOI:** 10.1186/s41199-016-0001-1

**Published:** 2016-06-03

**Authors:** Barbara Burtness

**Affiliations:** Department of Internal Medicine, Yale University School of Medicine and Yale Cancer Center, New Haven, Connecticut USA

The study of head and neck cancers in the clinic and in the laboratory has been radically transformed in recent years. The emergence of a subset of patients with Human Papillomavirus (HPV)-initiated oropharynx cancer, now representing the majority of new head and neck cancers at some sites in the United States, was followed by the recognition that these HPV-associated cancers represented a different disease [1]. HPV-initiated cancer not only has a different etiology, but also differs in treatment responsiveness, and biomolecular profile. Research on these more curable cancers increasingly will incorporate patient-reported outcomes and survivorship questions. At the same time, it has become clear that treatment advances have been only modest for HPV-negative cancers, and novel approaches to characterizing tumors and identifying therapeutic targets are urgently needed for these patients [2].

The advent of next-generation sequencing and publication of The Cancer Genome Atlas genomic profile in head and neck cancer have led to an initial classification of head and neck cancers into 4 intrinsic subtypes, with mutations in tumor suppressor genes the most common molecular abnormalities [3]. Ongoing efforts are likely to expand on these initial data with greater numbers of HPV-initiated cancers and cancers from non-smokers, as well as more detailed integrative analysis incorporating methylation and proteomic signatures. Successful targeted therapy of cancers driven by loss of tumor suppressor gene function is likely to require informatics approaches to identify potential synthetic lethal targets. The revolution in immuno-oncology is also relevant in head and neck cancer, with preliminary data indicating responsiveness to immune checkpoint inhibition for both HPV-associated and HPV-negative cancers [4].

The rapidity with which the field is moving, and the richness of these data sets, creates the need for a new journal dedicated to this field, one with diverse expertise across the Editorial Board, the ability to rapidly review submissions, and open access. We intend to publish original articles, reviews, case reports, and position papers across all disciplines that impact the direction of research and clinical care in head and neck cancer. Studies of standard and novel treatment approaches, including radiation, surgery, cytotoxic and targeted systemic therapy, vaccines and immunotherapy; translational science characterizing the biology of head and neck cancer subtypes, validation of prognostic and predictive biomarkers, and identification of novel therapeutics targets; virology of HPV and – for Epstein-Barr Virus (EBV)-associated nasopharynx cancer – EBV in the context of the head and neck; prevention strategies for those at risk for head and neck cancer; database analyses; and strategies to assess and improve functional outcome and quality of life, will be the core material published in *Cancers of the Head & Neck*.

An Editorial Board has been assembled consisting of 21 distinguished members from Europe, Asia and North America. Their expertise spans medical oncology, radiation oncology, head and neck surgery, head and neck pathology, virology, signaling, genomics, biomarkers, immuno-oncology, and survivorship.

The journal is published by BioMed Central (BMC)—an important leader in open access publishing, which currently publishes over 290 peer-reviewed open access journals. The strength of the BMC editorial offices will support our efforts to publish the highest quality papers after efficient and fair scientific peer review. *Cancers of the Head & Neck* operates a single-blind peer-review system aiming to provide authors with a first decision within 6 weeks and once accepted, to publish manuscripts online within 2 weeks. All articles in *Cancers of the Head & Neck* are freely and universally accessible online upon publication, so authors’ work is available to readers everywhere irrespective of the wealth of their institution. This ensures that authors’ work is disseminated to the widest possible audience and fosters the most active academic exchange [5].
